# Determination of Antibiotics Consumption in Buali-Sina Pediatric Hospital, Sari 2010-2011 

**Published:** 2014

**Authors:** Ebrahim Salehifar, Mohammadmehdi Nasehi, Gohar Eslami, Sima Sahraei, Reza Alizadeh Navaei

**Affiliations:** a *Department of Clinical Pharmacy, Faculty of Pharmacy, Thalassemia Research Center, Mazandaran University of Medical Sciences, Sari, Iran. *; b *Department of Pediatrics, Faculty of Medicine, Mazandaran University of Medical Sciences, Sari, Iran. *; c *Department of Pharmaceutical Care, Buali Sina Hospital, Mazandaran University of Medical Sciences, Sari, Iran. *; d *Clinical Research Development Center, Buali Sina Hospital, Faculty of Medicine, Mazandaran University of Medical Sciences, Sari, Iran. *

**Keywords:** Antibiotic overusage, Pediatrics, DDD-antibiotic consumption, Ceftriaxone

## Abstract

The increasing prevalence of antibiotic-resistant bacteria is a major health-care problem worldwide. WHO recommends DID (daily defined dose per 100 Inhabitant per day) as a standard tool for measurement of antibiotic consumption. Since there was not any information regarding the antibiotics usage pattern in the north of Iran, the aim of this study was determine this in our centre.

This cross-sectional study was performed in Buali Sina hospital. Using the health information system (HIS) database, records of patients hospitalized during 22 Sep 2010 – 21 Sep 2011. Data of different wards including Neonatal, NICU, PICU, Pediatrics and Pediatric surgery were separately extracted and analyzed. Drug consumption data were expressed as DID. SPSS 16 software was used for statistical analysis. Independent samples t-test was used to compare the quantitative variables.

A total of 4619 in-patients records during 1 year of study including 2494 patients in fall and winter and 2125 patients in spring and summer were evaluated. The most hospitalized patients were in Pediatric ward (43.9 %). The highest DID value were obtained for ceftriaxone (21.7), ampicillin (6.05) and vancomycin (4.7), while the lowest value was for gentamicin (0.01). In both cold and warm seasons, Ceftriaxone was the most frequent prescribed antibiotic.

The rate of antibiotics consumption especially Ceftriaxone in our setting was significantly higher than the other centers. Strategies for more justified administration of antibiotics especially broad –spectrum ones are necessary.

## Introduction

The increasing prevalence of antibiotic-resistant bacteria is a major health-care problem worldwide (1). The relationship between emergence of resistance and antibiotic use and misuse is well recognized (2). 

The overuse of antibiotics and poor compliance with infection control measures have been identified as the two major reasons for increasing antimicrobial resistance (1, 3, 4). Some evidences show that antibiotics are prescribed inappropriately in up to 50% (3, 5). Similar pattern of unnecessary antibiotics prescription was reported in children, especially by general practitioners (6). 

The total amount of an antibiotic used in a particular geographical area over a certain period of time is among of major causes of occurring antibiotic resistance (2, 7). 

Problems associated with the overuse of antibiotics include development of antibacterial resistance, raising the burden of chronic diseases, increasing costs of health services, and the development of side effects (*e.g*. gastrointestinal effects)(8).

The considerable amount of antibiotics misuse in children is one of the most important global public health issues (9).

Different methods of measuring antibiotic use are applied in the different studies (10-12). World Health Organization (WHO) recommends DID (daily defined dose per 100 bed-days Inhabitant per day) as a standard tool for measurement of antibiotic consumption in inpatient setting. Defined daily dose (DDD) is the average maintenance dose per day for a drug used in its main indication in adults (10, 12).

Knowledge of prescription patterns is an important tool in rational drug therapy. Children were subject to increasing exposure to antibiotics throughout the 1980s, so rational drug therapy is especially important for this age group (13). 

Previous studies have demonstrated a large variation in pattern of antibiotic use in different countries (14, 15). Since there was not any information regarding the antibiotics usage pattern in the north of Iran, this study was conducted to address this issue in our setting. 

## Experimental

*Setting*

This cross-sectional study was performed in Buali-Sina hospital; an educational university- affiliated hospital consisted of 6 wards with 220-beds, in north of Iran, Sari. The inclusion criteria were all of the neonates, infants and pediatrics who hospitalized during 22 Sep 2010 – 21 Sep 2011 and received any antibiotics. 

Records of patients were studied by using the health information system (HIS) database. Data of fall and winter seasons (22 Sep 2010 – 19 Mar 2011) was separately gathered and analyzed from spring and summer seasons (20 Mar 2011 – 21 Sep 2011). Data of different wards including Neonatal, NICU, PICU, Pediatrics and Pediatric surgery were separately extracted and analyzed. 

Drug consumption data were expressed as defined daily doses (DDD) per 100 inhabitants per day (DID). In order to calculate DID, ATC (Anatomical Therapeutic Chemical) codes and DDD for each antibiotic were obtained from WHO website (http://www.whocc.no/atc_ddd_index/?code=J01DH51 ; ATC/DDD Index 2012; last updated: 2011-12-19; accessed: 2012/11/05) (16). The following formula was used to calculate DID (17).

*DDD per100 inhabitant per day (DID) =(Total consumption in DDDs x 100)/(Covered inhabitants x Days in the period of data collection*).

*Statistical analysis*

The HIS data was transferred to Excel program for calculation of DID. SPSS 16 software was used for statistical analysis. Independent samples t-test was used to compare the quantitative variables including “days of hospitalization”, and “amount (mg) of antibiotics used” between two time periods. 

p-value less than 0.05 were considered as a significant difference. 

## Results

A total of 4619 in-patients records during 1 year of study including 2494 patients in fall and winter seasons and 2125 patients in spring and summer seasons were evaluated. 

The numbers of hospitalized patients in different wards were showed in Table 1. During 1 year of study, most hospitalized patients were in Pediatric ward (43.9 %) followed by Surgery, Neonate, NICU and PICU wards.

**Chart 1:**
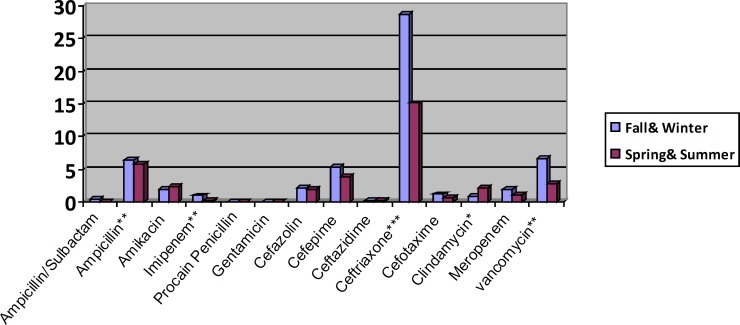
Antibiotics consumption based on DDD/100 inhabitant/day p-value: comparison of mean differences of mg used of antibiotics in different time periods (*e.g*., Fall & Winter vs. Spring &Summer), obtained by independent samples t-test. *, ** and *** display p-value<0.05, <0.01 and <0.001 respectively.

The result of DID (DDD/100 inhabitant/day) calculation for each antibiotic in cold and warm seasons was demonstrated in Chart 1. 

The highest DID value were obtained for ceftriaxone (21.7), ampicillin (6.05) and vancomycin (4.7), while the lowest value was for gentamicin (0.01). 

**Table 1:** Bed-days of hospitalized patients in different wards

**p-value**	**Total**			**Spring & Summer**			**Fall & Winter**			**Hospital** **wards**
	**Bed-days**	**Day-Stay (Mean±SD)**	**Number of patients(%)**	**Bed-days**	**Day-Stay (Mean±SD)**	**Number of patients(%)**	**Bed-days**	**Day-Stay (Mean±SD)**	**Number of patients (%)**	
	9101	14.4 ± 13	632(13.7)	5470	15.54 ± 14.7	352(16.6)	3612	12.9 ± 10.3	280(11.2)	**Neonatal**
	21329	21.35 ± 19.3	999(21.6)	12490	24.3 ± 23.3	514(24.2)	8847	18.24 ± 13.2	485(19.4)	**NICU**
	8951	15.9 ± 17.9	563(12.2)	5059	20.4 ± 24.1	248(11.7)	3875	12.3 ± 9.4	315(12.6)	**PICU**
-	5179	6.3 ± 6.3	822(17.8)	2225	5.98 ± 6.2	372(17.5)	2952	6.56 ± 6.3	450(18)	**Surgery**
-	11862	7.4 ± 7.5	1603(34.7)	4582	7.17 ± 8.8	639(30.1)	7269	7.54 ± 6.5	964(38.7)	**Pediatrics**
0.66	56422	12.2 ± 14.3	4619(100)	29826	14.03 ± 17.9	2125(100)	26555	10.66 ± 9.96	2494(100)	**Total**

p-value: mean differences of days stay in the different wards between hot and cold seasons obtained by independent samples t-test .

* , ** and *** display p-value<0.05, <0.01 and <0.001 respectively.

Some antibiotics have significant different consumption in hot and cold seasons. For example, ampicillin and clindamycin were significantly more prescribed in spring and summer seasons, while vancomycin, ceftriaxone and imipenem were more frequently prescribed in fall & winter seasons. The total amount of antibiotics used was not significantly different in cold and hot seasons. 

Considering the percent of patients received each antibiotic, ceftriaxone (44.8%), amikacin (14.8%) and ampicillin (14.5%) were the most common ones, while the least one was penicillin (0.2%) (Chart 1). 

In both cold and warm seasons, Ceftriaxone was the most frequent prescribed antibiotic (Chart 1, Table 2). 

Antibiotic usage in different wards of the hospital including NICU, PICU, Pediatric, Neonate and Surgery was shown in Table 2. Mean differences of day-stay in neonatal wards, NICU and PICU were significantly different between hot and cold seasons. 

In both cold and warm seasons, Ceftriaxone was the most frequent prescribed antibiotic. In all different wards apart from NICU and neonatal wards, ceftriaxone had a highest DID level, whereas ampicillin was associated with the highest amount of DID level in both of them (Chart 1 and Table 2). 

**Table 2:** Antibiotics consumption in different wards based on DID

Neonate			Surgery			NICU			PICU			***Pediatrics***			
**Total (P-value)**	**Spring & Summer**	**Fall & Winter**	**Total (P-value)**	**Spring & Summer**	**Fall & Winter**	**Total (P-value)**	**Spring & Summer**	**Fall & Winter**	**Total (P-value)**	**Spring &Summer**	**Fall & Winter**	**Total (P-value)**	**Spring &Summer**	**Fall & Winter**	**Antibiotic** ** (ATC code)**
0.08(-)	0	0.18	0(-)	0	0	0.06(-)	0	0.24	1.2 (-)	0	1.91	0.20(-)	0	0.34	**Amipicillin/Sulbactam (J01CR01)**
12.97(-)	11.92	14.3	1.61(***)	1.14	2.06	13.98(-)	9.31	28.79	3.33(-)	2.7	3.68	1.21(-)	1.11	1.28	**Ampicillin (J01CA01)**
3.1(-)	3.3	2.8	1.38(-)	1.25	1.47	4.24(-)	3.04	8.04	3.17(-)	4.41	2.5	0.72(-)	1.25	0.38	**Amikacin(J01GB06)**
0.12(-)	0.15	0.08	0.14(-)	0.19	0.08	0.53(-)	0.44	0.81	1.35(-)	0.79	1.67	1.01(-)	0.13	0	**Imipenem (J01DH51)**
0(-)	0	0	0.02(-)	0.05	0	0(-)	0	0	0.012(-)	0	0.02	0.06 (**)	0.04	0.078	**Procaine Penicillin (J01CE09)**
0(-)	0	0	0.03(-)	0.04	0.023	0.002(-)	0.003	0	0.06(-)	0.16	0	0.01(-)	0.02	0	**Gentamicin (J01GB03)**
0.009(-)	0	0.02	3.71(**)	2.5	4.85	0(-)	0	0	1.62(-)	1.89	1.46	3.45(-)	4.87	2.550	**Cefazolin (J01DB04)**
0.92(-)	0.97	0.85	0.4(-)	0	0.79	1.32(-)	1.16	1.83	19.82(-)	28.2	15.033	6.74(-)	5.75	7.379	**Cefepime (J01DE01)**
0(-)	0	0	0.78(-)	0.77	0.79	0(-)	0	0	0.41(-)	1.13	0	0.15(-)	0.137	0.16	**Ceftazidime(J01DD02)**
0.09(-)	0.085	0.097	32.3(**)	25.1	39.2	0(-)	0	0	29.6(-)	37.8	24.83	36.9(**)	30.42	41.18	**Ceftriaxone(J01DD04)**
1.41(**)	0.99	1.96	0.6(-)	0.4	0.79	0.92(-)	0.37	2.7	2.48(-)	2.67	2.38	0.56(-)	0.66	0.49	**Cefotaxime (J01DD01)**
0(-)	0	0	0.58(-)	1.18	0	0(-)	0	0	8.68(-)	14	5.6	1.87(-)	3.81	0.6	**Clindamycin(J01FF01)**
0.53(-)	0.52	0.54	0.56(-)	0.38	0.73	1.47(-)	1.03	2.85	9.59(-)	9.93	9.4	0.61(-)	0.23	0.9	**Meropenem (J01DH02)**
1.02(-)	1.18	0.83	1.97(-)	1.24	2.67	2.15(-)	1.32	4.8	13.75(-)	19.3	10.6	7.07(*)	3.04	9.72	**Vancomycin(J01XA01)**
20.23	19.10	21.67	44.08	34.25	53.40	24.65	16.65	50.014	95.05	122.982	79.04	60.60	51.47	64.99	**Total**
1.45	1.36	1.55	3.14	2.44	3.81	1.76	1.18	3.57	6.78	8.78	5.65	4.32	3.67	4.65	**Mean**

P-value: comparison of mg used of each antibiotic in different time periods (*e.g.,* Fall & Winter vs. Spring &Summer), obtained by independent samples t-test.

* , ** and *** display p-value<0.05, <0.01 and <0.001 respectively.

## Discussion

Our study shows overuse of antibiotics in our centre. Total DID of antibiotic usage is 46.32 DDD/100inh/day and some antibiotics like ceftriaxone and ampicillin have a very high DID numbers.

One of the advantages of our study is the comparison of antibiotic consumption in two different time periods (fall & winter Vs spring & summer). The result of this study demonstrates more admission and hospitalization days in neonatal, NICU and PICU wards in fall and winter. This may be related to the higher incidence of severe infectious diseases in the cold seasons, therefore more admission occurs in intensive care units. 

Vancomycin, ceftriaxone and imipenem were more frequently prescribed in fall & winter seasons. It may be associated with more severe infectious disease and more resistant antibiotical pattern in cold seasons.

Several studies have shown the antibiotic utilization pattern in various hospitals around the world (18, 19). Systemic antibiotics were by far the most widely used drugs in children (20, 21). However, children differ from adults regarding pharmacokinetics and pharmcodynamics (6, 20, 22). 

Different studies have been done on the pattern of antibiotics use in pediatric patients that has shown in Table 3. This shows the comparison of most common used antibiotics and DID number in different studies.

**Table 3:** The comparison of most common used antibiotics and DID number in different studies.

**Different ** **studies**	**Present** **study**	**Italy,** **2009 [23]**	**Denmark, ** **2009 [23]**	**Croatia, 2000[24]**	**Russia, ** **2000 [24]**	**Italy,** **2000[6]**	**Libya, 2008[17]**	**Libya , 2009[17]**
Total inhabitants	4207	22469 (during1year)	47173 (during1year)	6497 (during1year in six departments)	5467 (during1year in six departments)	215696	100 (during 15 months)	100 (during 15 months)
Study setting	inpatient	outpatient	outpatient	inpatient	inpatient	outpatient	inpatient	inpatient
Most common used antibiotics: (DDD/100inh/day Or percentage)	Ceftriaxone (21.7) Ampicillin (6.05)	Amoxicillin+ enzyme inhibitor (2.3) Amoxicillin (1.4)	Phenoxymethyl- Penicillin (1.27) Amoxicillin (0.7)	Cefuroxime (6.8) Ceftriaxone (5)	Amoxicillin (2.6) Ampicillin (1)	Amoxicillin+ enzyme inhibitor (27%) Amoxicillin (26%)	Amoxicillin+ enzyme inhibitor (0.52) Cefixime (0.46) Cefotaxime (0.46)	Cloxacillin (0.9) Ampicillin (0.9) Amoxicillin+ enzyme inhibitor (0.65)
Total antibiotics DDD/100inh/day :	46	6.7	3.5	29	8.3	119	3	6.4

Krivoy N, *et al. *(2007) has reported concern about the continuous and excessive use of antimicrobial agents that cause the emergence of antibiotic-resistant organisms. Evaluating antibiotic prescription and monitoring of antimicrobial uses are strategies recommended for management of resistance to antimicrobials in hospitalized patients. Also several studies report Antimicrobial resistance raises already-rising health care costs and increases patient morbidity and mortality(17, 25).

In our study, the most frequently used antibiotics were ceftriaxone 46.85%, ampicillin 13%, vancomycin 10.1%, cefepime 9%, amikacin 4.6%, and cefazolin 4%.

In the study of Katakam, *et al. *in pediatrics ward, Amoxicillin+Clavulanic acid (37%) and ampicillin (18%) were the most frequent prescribed antibiotics (17). 

The least frequently used antibiotic was gentamicin in all duration of study. Due to concern regarding the resistance to Gentamicin, the high usage of amikacin in our center could be explained. In contrast to our study, Thrane *et al*. reported that penicillins were the most common antibiotics prescribed (26). 

Drug consumption data were expressed as defined daily doses (DDD) per 100 inhabitants per day (DID). The highest value of ceftriaxone may imply that it was a choice as an empiric therapy in our hospital. In Katakam, *et al*., study ceftriaxone DDD/1000/Day was 2.6 in 2008 and 4 in 2009(17). In Resi, *et al. *study the most frequently used antibiotics were cephalosporins group (43.7% of treated children) and ceftriaxone was only 2.9% of total antibiotic used (6). Also, in our centre cephalosporins (63.7%) were the most prescribed antibiotics group but it was higher than other studies reports.

overuse of cephalosporins specially broad spectrum ones can cause the emergence of antimicrobial resistance and the overgrowth of pathogenic microorganisms(27, 28).

The present study demonstrated overuse of ceftriaxone apart from in neonate patients. Overuses and misuse of ceftriaxone and cefepime in our study, may lead to increases in antibiotic resistance. 

According to the Gagliotti, *et al. *study, the most frequent antibiotic between 0-23 month years old was cephalosporin, but ceftriaxone had been prescribed in only 2.02% of all patients which is significantly lower than the results we have achieved in the present study(27). 

According to the adverse effects of ceftriaxone especially in hyperbilirubinemic neonates and preterm babies, great potential for bilirubin displacement and developing kernicterus, the limited use of ceftriaxone in neonatal patients is predictable (29, 30). 

## Conclusion

The rate of antibiotics consumption in our setting was significantly higher than the other centers. 

The high usage of Ceftriaxone, as a valuable third generation cephalosporin, was a prominent finding. 

Strategies for more justified administration of antibiotics especially broad –spectrum ones are necessary.

*Limitation*

We measured antibiotics usage with adult DDDs number. It is notable that the previous studies in pediatrics do not estimate the DDD number in this population, so the methodology of our study were similar to other studies in this field. 
